# Machine Learning-Based Virtual Screening for the Identification of Novel CDK-9 Inhibitors

**DOI:** 10.3390/biom16010012

**Published:** 2025-12-20

**Authors:** Lisa Piazza, Clarissa Poles, Giulia Bononi, Carlotta Granchi, Miriana Di Stefano, Giulio Poli, Antonio Giordano, Annamaria Medugno, Giuseppe Maria Napolitano, Tiziano Tuccinardi, Luigi Alfano

**Affiliations:** 1 Department of Pharmacy, University of Pisa, 56126 Pisa, Italy; lisa.piazza@phd.unipi.it (L.P.); giulia.bononi@unipi.it (G.B.); carlotta.granchi@unipi.it (C.G.); miriana.distefano@farm.unipi.it (M.D.S.); giulio.poli@unipi.it (G.P.); 2 Genomics and Experimental Medicine Program, Scuola Superiore Meridionale (SSM, School of Advanced Studies), Via Mezzocannone 4, 80078 Napoli, Italy; c.poles@tigem.it; 3 Telethon Institute of Genetics and Medicine, Via Campi Flegrei 34, 80078 Napoli, Italy; 4 Sbarro Institute for Cancer Research and Molecular Medicine, Center for Biotechnology, College of Science and Technology, Temple University, Philadelphia, PA 19122, USA; antonio.giordano@temple.edu; 5 Department of Medical Biotechnologies, University of Siena, 53100 Siena, Italy; a.medugno1@studenti.unisi.it; 6 Clinical and Translational Oncology Program, Scuola Superiore Meridionale (SSM, School of Advanced Studies), University of Naples Federico II, 80131 Napoli, Italy; 7 Consorzio Interuniversitario Nazionale per la Scienza e Tecnologia dei Materiali (INSTM), 50121 Firenze, Italy; 8 Department of Breast and Thoracic Oncology, Istituto Nazionale Tumori-IRCCS-Fondazione G. Pascale, 80131 Napoli, Italy; l.alfano@istitutotumori.na.it

**Keywords:** cyclin-dependent kinase 9, machine learning, cancer therapy, drug discovery, virtual screening

## Abstract

Cyclin-dependent kinase 9 (CDK9) is a key regulator of transcriptional elongation and DNA repair, supporting cancer cell survival by sustaining the expression of oncogenes and anti-apoptotic proteins. Its overexpression in multiple malignancies makes it an attractive target for anticancer therapy. Here, we report a machine learning (ML) based approach to identify novel CDK9 inhibitors. Through systematic data collection and preprocessing, seventy predictive models were developed using five algorithms, two classification settings, and seven molecular representations. The best-performing model was employed to guide a virtual screening (VS) campaign, resulting in the identification of 14 compounds promising for their potential inhibitory effect. Upon enzymatic assays, two molecules with inhibitory activity in the low micromolar range were selected as promising candidates and further tested in three cancer cell lines with distinct genetic backgrounds. These experiments led to the identification of a novel compound exhibiting interesting therapeutic potential, both as a single agent and in combination with Camptothecin (CPT), revealing varying response profiles across the tested cell lines. These results illustrate the power of integrating ML within anticancer drug discovery pipelines and represent a valuable starting point for the development of novel CDK9 inhibitors.

## 1. Introduction

Protein kinases are essential regulators of intracellular signaling, controlling a broad spectrum of cellular processes, including proliferation, differentiation, and apoptosis, through the phosphorylation of specific substrates. Cyclin-dependent kinases (CDKs) form a well-characterized subfamily of serine/threonine kinases, whose activation is tightly regulated by the binding of specific cyclin proteins. CDKs are classically divided into two functional groups: those primarily involved in cell cycle control (e.g., CDK1, CDK2, CDK4, and CDK6), and those that regulate gene transcription through RNA polymerase II (e.g., CDK7, CDK8, CDK9, CDK12, CDK13, and CDK19) [1]. More recently, the role of transcription-associated CDKs in oncogenesis has gained increasing attention. Among these, CDK9 is a multifunctional kinase regulating many different processes from DNA repair to transcriptional elongation [2]. In complex with cyclin T1, it forms the positive transcription elongation factor b (P-TEFb), which phosphorylates the C-terminal domain (CTD) of RNA polymerase II, as well as the elongation factors DSIF (DRB sensitivity-inducing factor) and NELF (negative elongation factor). This phosphorylation event is crucial for overcoming the promoter-proximal pausing that occurs shortly after transcription initiation, a process known as the early elongation checkpoint. Without CDK9 activity, RNA polymerase II remains stalled, and productive elongation is impaired, resulting in incomplete transcript synthesis [1,3,4]. CDK9 activation itself depends on phosphorylation of a conserved threonine residue (T186) within the T-loop, mediated by autophosphorylation or upstream kinases such as CDK7, which stabilizes the active conformation of the enzyme [4].

Given this central role in transcriptional control, CDK9 is frequently exploited by cancer cells to sustain the expression of oncogenes and short-lived anti-apoptotic proteins such as MYC and MCL-1. Its overexpression has been observed in several solid and hematological malignancies, including pancreatic, ovarian, endometrial, and colorectal cancers, where it is associated with poor prognosis, enhanced metastatic potential, and resistance to conventional therapies [5,6]. In addition to the predominant exploration for cancer treatment, CDK9 inhibition has gained relevance in a broader spectrum of medical areas suffering significant unmet needs, such as autoimmune, inflammatory, and cardiac diseases [7,8], further underscoring the therapeutic promise of CDK9.

Nevertheless, a straightforward clinical translation remains hampered by a narrow therapeutic window and poor clinical response [9]. These issues strengthen the need for fast and advanced approaches to identify novel CDK9 inhibitors with improved pharmacological profiles.

In recent years, computational approaches have emerged as powerful tools to complement traditional drug discovery. In silico methods such as molecular docking, pharmacophore modeling, and machine learning (ML) offer efficient means to predict compound–target interactions, reduce screening costs, and accelerate lead identification. Among these, ML techniques have demonstrated notable success in modeling complex structure-activity relationships, enabling the prioritization of promising molecules as novel potent kinase inhibitors from large chemical libraries with increased accuracy [10,11,12]. In this study, we applied an ML-driven virtual screening (VS) strategy to identify novel small molecules acting as inhibitors of CDK9. Curated bioactivity datasets, molecular fingerprints, and advanced classification models were integrated to predict inhibitory potential. Top-ranking candidates resulting from VS were first selected and subjected to enzymatic assays to experimentally validate their predicted inhibitory activity toward CDK9. Among them, the two most promising compounds were further evaluated in cell-based assays, leading to the identification of a novel CDK9 inhibitor displaying significant cytotoxic activity. Finally, this compound was investigated through molecular docking and molecular dynamics (MD) simulations to characterize its interaction with CDK9, and to propose a plausible binding mode within the protein active site. Notably, this analysis also represents a valuable contribution to support future CDK9 studies by providing a solid structural rationale for subsequent optimization efforts. To further enhance the practical usability of the developed model, we made it publicly available through a dedicated GitHub repository (https://github.com/MMVSL/CDK9Screen, accessed on 25 October 2025), thereby providing the scientific community with a transparent, accessible, and easy-to-use computational tool to predict the potential inhibitory activity of candidates of interest against CDK9.

## 2. Materials and Methods

### 2.1. Data Processing

The ChEMBL database [13] (version 31) was chosen as the primary activity data repository. Compounds with reported inhibitory activity against CDK9, either as a single protein (target ChEMBL ID: CHEMBL3116, 2213 entries) or complexed with cyclin T1 (target ChEMBL ID: CHEMBL2111389, 2380 entries), were retrieved and processed through the following filtering pipeline. Firstly, only activity records with a standard relation type of “=” (i.e., exact values) for IC_50_, EC_50_, *K*_i_, or *K*_d_ were retained. Entries with missing SMILES (Simplified Molecular Input Line Entry System) or unspecified activity values were discarded. Since IC_50_ data represented approximately 80% of the available measurements, we decided at this stage to prioritize these entries for model development, thus using them as training compounds, while reserving records with EC_50_, *K*_i_, and *K*_d_ values as test set compounds for model evaluation. In light of this, when multiple measurements were available for the same compound, the activity expressed as IC_50_ was preferred in order to enrich the training set. This procedure resulted in two preliminary datasets comprising 1914 compounds for the training set and 464 for the test set.

Subsequently, both the preliminary training and test sets underwent duplicate instance removal and structural standardization, including salt elimination and ionization at physiological pH using the software MolBook Pro [14,15]. This preprocessing pipeline resulted in the identification of 1629 final compounds designated for the training set (the distribution of their activity values is reported in Figure S1) and 372 compounds for the test set.

Two classification approaches were then defined to distinguish active from inactive compounds, resulting in two separate classification schemes. Each approach was applied independently to both the training and test sets, generating a total of four datasets, one classified train/test pair for each classification scheme. According to the first approach (Lead-Oriented, LO), compounds with activity ≤1 µM, which corresponds to p*Activity* ≥ 6, were labeled as active, while those with an activity ≥5 µM were labeled as inactive (p*Activity* ≤ 5.30). These thresholds were derived from a prior study conducted on CDK5, a kinase from the same protein family for which this cutoff proved to be effective [11].

A second classification scheme (Potency-Oriented, PO) was defined to emphasize compounds with higher potency. In this case, molecules with activity ≤31.6 nM, which corresponds to p*Activity* ≥ 7.5, were labeled as active, while those with activity ≥3.2 µM, corresponding to p*Activity* ≤ 5.5, were categorized as inactive. In each classification scheme, compounds with activity values falling within the two classification thresholds were excluded from the corresponding dataset.

As a result, the classified training set for the PO scheme contained 565 molecules, of which 447 were classified as active and 118 as inactive. The training set of the LO approach instead included a total of 1465 compounds (1375 classified as active; 90 classified as inactive). Similarly, the corresponding test sets comprised 150 compounds for the PO scheme (106 labelled as active and 44 labelled as inactive) and 309 compounds for the LO scheme (279 marked as active; 30 marked as inactive).

Subsequently, to mitigate the pronounced imbalance toward the active class observed across all four datasets, additional inactive compounds were incorporated from the ChEMBL 31 database. These entries corresponded to records in which the reported activity was expressed with the relation symbol “>”, meaning that the actual activity value was higher than the one reported. During the initial data processing, such compounds had been excluded because this format did not provide a precise numerical value. Once the activity thresholds were established, however, compounds reported with a “>” relation were confidently assigned to the inactive class whenever their recorded activity exceeded the predefined inactivity cutoff. Precisely, 228 and 62 inactive compounds were added to the training and test sets of the PO approach, respectively.

Within the LO scheme, instead, this step resulted in the integration of 224 inactive molecules in the training set, and 246 in the test set. Despite this first integration, the datasets remained unbalanced. Therefore, an additional set of inactive decoys was retrieved from the ZINC20 database [16], as previously performed [12]. All decoy compounds underwent the same data processing and standardization protocol described previously to guarantee consistency across the integrated datasets. Additionally, a control procedure was performed to ensure that no instances were in common between each train and the corresponding test set, preserving the integrity of the model evaluation process. As a result, four curated datasets were obtained, each perfectly balanced in terms of class distribution; i.e., LO training and test sets containing 2750 and 558 compounds, respectively, and the corresponding PO datasets with 894 (training) and 212 (test) instances.

### 2.2. Molecular Representations

To enable ML algorithms to interpret chemical structures and detect relevant features to make accurate predictions, molecules must first be translated into numerical representations. In this study, we explored seven types of molecular encodings: three established fingerprints (RDKit, Morgan, and PubChem fingerprint), a set of 208 numerical descriptors derived using RDKit, and three novel hybrid representations obtained by the concatenation of each fingerprint with descriptors. All molecular representations were generated starting from the SMILES strings of each compound, exploiting functionalities included in the RDKit Python library (version 2024.3.5) [17].

*RDKit fingerprint*: this representation captures molecular structure by identifying various connectivity patterns, known as subgraphs, that correspond to atom-bond paths of different lengths within a molecule. Each subgraph is then encoded into a specific position in a binary vector through a hashing process. RDKit fingerprints were generated using the RDKit library with a vector length of 1024.

*Morgan fingerprint*: this method encodes structural information by iteratively analyzing the neighborhood of each heavy atom up to a defined radius. In this study, we set a radius of two bonds. Each local atomic environment is then translated into a numerical identifier through a hashing technique. Coherently with RDKit fingerprints, Morgan fingerprints were computed with a fixed vector length of 1024 bits using the RDKit toolkit.

*PubChem fingerprint*: this method employs a predefined dictionary of chemical features. Each molecule is screened for the presence of 881 specific substructures, with a corresponding binary vector used to record their presence (1) or absence (0).

*RDKit Descriptors*: these numerical features reflect various chemical and physical properties of a molecule. Using RDKit’s built-in descriptor functions, we calculated 208 attributes per compound and normalized them using Scikit-learn Min-Max scaler [18] to ensure uniform magnitude across the dataset.

*Hybrid representations*: these representations were generated by concatenating the descriptors with the Morgan (HybridMR), PubChem (HybridPR), or RDKit (HybridRR) fingerprint, respectively.

### 2.3. Machine Learning Algorithms

ML models were developed using two primary Python frameworks: Scikit-learn [18] for traditional algorithms and TensorFlow for neural network-based approaches. All models adopted a supervised classification strategy, designed to discriminate between compounds with potential inhibitory activity against CDK9 and those considered inactive. The output of each model consists of a probability score (PS) indicating the likelihood that a given compound functions as an active inhibitor. To build the classification models, five distinct algorithms were employed: Random Forest (RF), Support Vector Machine (SVM), k-Nearest Neighbors (KNN), Gaussian Process (GP), and Multi-Layer Perceptron (MLP). For each algorithm, different hyperparameters were optimized to find the most robust architecture setup.

RF combines a multitude of decision trees, each trained on a random subset of the training data sampled with replacement. The predictions of all trees are eventually aggregated through majority voting to determine the final output. The most relevant hyperparameters optimized for RF models were *n_estimators*, which specifies the total number of decision trees in the ensemble (with tested values 100 and 500), and *max_features*, which defines how many features are randomly selected at each node to identify the best split (with tested values *None*, *sqrt*, and *log2*).

SVM aims to identify an optimal hyperplane that best separates the two classes in a high-dimensional feature space. This space is implicitly defined through kernel functions, which enable the algorithm to compute inner products in the feature space without explicitly performing the transformation, an approach known as the kernel trick. As a result, all computations are carried out in the input space, making the method both efficient and scalable to complex, non-linear classification problems. The kernel functions explored during hyperparameter tuning were Scikit-learn available kernels such as *linear*, *poly*, *rbf*, *sigmoid*, and *Tanimoto* [19].

KNN classifies data points based on the majority label among the k closest training instances, using a distance metric to determine proximity. The number of neighbors to consider (with tested values being odd integers ranging from 3 to 15) and the option to weight neighbors based on their distance to the query compounds were included as tunable hyperparameters.

GP represents a non-parametric probabilistic framework that uses kernel functions to capture complex patterns in the data and estimate the likelihood that a sample belongs to a certain class. Different Scikit-learn kernel functions were evaluated during optimization, including *DotProduct*, *RationalQuadratic*, *Matern*, *WhiteKernel*, and *RBF*.

MLP is part of the deep learning family. This architecture consists of an input layer, one or more hidden layers, and an output layer. Each hidden layer contains multiple neurons, where each neuron applies a weighted sum of its inputs followed by a non-linear activation function, such as ReLU. This setup allows MLP to model complex, non-linear relationships within the data. The hidden layer size and the batch size were among the main optimized hyperparameters. For the batch size, the tested values were 100 and 200. For the hidden layer size, instead, all the possible combinations between (2, 3) layers and (100, 200, 1000) neurons in each layer were tested.

### 2.4. Model Evaluation and Structural Similarity Metrics

To evaluate model performance, we focused on three evaluation metrics: Matthews correlation coefficient (MCC) [20], precision, and recall. Their formulas are herein reported.

$$MCC=\frac{\left(TP\cdot TN\right)-\left(FP\cdot FN\right)}{\sqrt{\left(TP+FP\right)\left(TP+FN\right)\left(TN+FP\right)\left(TN+FN\right)}}$$

$$Precision=\frac{\mathrm{TP}}{\mathrm{TP}+\mathrm{FP}}$$

$$Recall=\frac{TP}{TP+FN}$$

True Positives (TP) and true negatives (TN) denote the correctly predicted active and inactive compounds, respectively. In contrast, false positives (FP) are inactive molecules that the model incorrectly identifies as active, while false negatives (FN) are active compounds that the model fails to recognize, misclassifying them as inactive.

MCC provides a comprehensive measure of classification quality by incorporating all four confusion matrix outcomes. It ranges from −1 to 1, where 1 indicates perfect prediction accuracy with no FP or FN, and −1 corresponds to completely incorrect predictions. Precision measures the proportion of correctly predicted active compounds among all predicted actives, ranging from 0 to 1, with higher values indicating fewer false positives. It reflects the reliability of positive predictions but does not account for false negatives. On the other hand, recall quantifies the model’s ability to identify all true active compounds, and it also ranges from 0 to 1.

In addition to these evaluation metrics, the Tanimoto coefficient [21] was used to assess molecular similarity. This measure compares molecular fingerprints of two compounds and yields values between 0 and 1, with values closer to 1 indicating higher structural similarity. The formula for Tanimoto similarity is herein reported:

$$T=\frac{s}{a+b-s}$$

where *s* is the number of shared bits set to 1 in both fingerprints, while *a* and *b* represent the total number of bits set to 1 in the fingerprint of the first and second molecule, respectively.

### 2.5. Model Development and Assessment

For each classification scheme, 35 ML models were derived by systematically combining 7 molecular representations with 5 ML algorithms. For classical ML approaches, namely RF, SVM, GP, and KNN, each model underwent a hyperparameter optimization phase using Scikit-learn’s Grid Search [18]. This method systematically explores predefined hyperparameter combinations through cross-validation (CV) to identify the best configuration based on the MCC, used here as the primary performance metric. Following tuning, each model was further evaluated through 5-fold CV to ensure robustness and consistency across different data splits. For every fold, the training data were randomly partitioned into 80% for model training and 20% for internal validation. An analogous workflow was applied to MLP-based models using a custom pipeline implemented in TensorFlow, adapting hyperparameter tuning and validation steps to neural network architectures.

Once the optimal parameters were identified, the final models were retrained on the complete training set and evaluated against the independent test set to assess their generalization capability.

All training procedures were executed on a workstation equipped with an Intel Core i7-12700K CPU, using 16 cores for parallelized operations where supported.

### 2.6. Virtual Screening

The VS library was generated starting from the Aldrich Market Select (AMS) 2022 catalogue. Compounds were curated using a standardization protocol consistent with the data processing steps applied to training and test sets, including salt removal and ionization at physiological pH. As a result, we obtained a final VS dataset containing a total of 7,722,826 commercial and properly standardized compounds.

The VS was performed on a workstation equipped with an Intel Core i7-12700K CPU.

### 2.7. Clustering

To select a structurally diverse subset of high-confidence candidates for in vitro testing, hierarchical agglomerative clustering was applied to the VS hits using Orange3 software version 3.36.0 [22]. In this method, each compound initially constitutes a separate cluster. Clusters are then iteratively merged based on pairwise distances, progressively assembling the most similar ones until all compounds are integrated into a single hierarchical structure. Compounds were encoded as RDKit fingerprints, and the similarity between compounds was measured using the Tanimoto coefficient computed via the RDKit package version 2024.3.5. Distance was then defined as one minus the Tanimoto similarity. Clusters were merged based on the average linkage criterion, which defines the distance between two clusters as the mean pairwise distance between their members.

### 2.8. Experimental Validation

#### 2.8.1. Enzymatic Assays

The inhibitory activity of the compounds identified by the VS was evaluated against CDK9/cyclin T1 using the fluorescence-based ADAPTA kinase assay (Thermo Fisher Scientific, Madison, WI, USA). The assay quantifies ADP generated during the kinase reaction through a competitive immunoassay employing a TR-FRET detection system. In the presence of an inhibitor, ADP formation by the kinase is reduced, leading to preservation of the antibody–tracer complex and a corresponding increase in the TR-FRET signal. Inhibitory potency was expressed as IC_50_ values, determined from logistic dose–response curves with 10 concentration points, each representing the average of two independent experiments.

#### 2.8.2. Immunofluorescence Analysis

HeLa cells were fixed in 4% paraformaldehyde and subsequently permeabilized with 0.2% Triton X-100. Blocking was performed with 1% bovine serum albumin (BSA) for 10 min at room temperature. Cells were then incubated for 1 h at RT with primary antibodies targeting: γH2AX Ser139 (1:200, ab2893, Abcam, Cambridge, UK) or pRPA32 S4/8 (1:200, A300-245A, Bethyl Laboratories, Montgomery, TX, USA). Corresponding secondary antibody conjugated with AlexaFluor 647 donkey anti-rabbit IgG (H + L) was applied. Samples were mounted using ProLong™ Gold Antifade Mountant with DAPI (Thermo Fisher Scientific). High-resolution immunofluorescence imaging was performed on a Zeiss LSM 900 Airyscan2 confocal microscope equipped with a Plan-Apochromat 63×/1.4 NA oil immersion objective. Image acquisition and processing utilized Zen 3.9 software Zeiss, Jena, Germany). Quantification of fluorescent foci per nucleus was conducted using Fiji (ImageJ) software, version 2.14.0/1.54f.

#### 2.8.3. Cell Viability Assays

HeLa, MCF-7, and MDA-MB-231 cells were seeded in triplicate into 96-well plates at densities of 1500, 3500, and 3000 cells per well, respectively, and allowed to adhere for 24 h. After 72 h of incubation, cells were fixed with 10% (*v*/*v*) trichloroacetic acid (TCA) and subsequently stained with 0.4% (*w*/*v*) sulforhodamine B (SRB) dissolved in 1% (*v*/*v*) acetic acid. Cell viability was quantified and expressed as a percentage relative to untreated control cells, which were normalized to 100%. Data represent the mean ± standard deviation (SD) from three independent experiments (n = 3). Statistical significance was determined using one-way ANOVA or Student’s *t*-test, followed by appropriate multiple-comparison post hoc tests to evaluate differences between experimental groups.

### 2.9. Molecular Docking Studies

Molecular docking was performed with GOLD 5.1 using the GoldScore fitness function and the X-ray structure of CDK9 in complex with a co-crystallized ligand (PDB ID: 6GZH [23]). The binding pocket was defined as the region encompassing all residues located within 15 Å from the co-crystallized ligand. While the option for early termination of runs was disabled, ring corner flipping was permitted, and 100 genetic algorithm runs were executed. All other parameters were instead kept at the default configuration [24]. The obtained docking solutions were clustered using an RMSD threshold of 2.0 Å. Subsequently, clusters representing at least 5% of the poses were considered, and their representative conformations extracted. Among them, only poses forming at least one hydrogen bond with the hinge region of the protein were retained for further investigations.

### 2.10. Molecular Dynamics Simulation

MD simulation was carried out using AMBER version 24 [25] employing an NVIDIA RTX 3080 GPU. The protein was modeled with the ff14SB force field, while ligands were parameterized using GAFF, and their partial charges assigned through the AM1-BCC method with the Antechamber module. The protein–ligand complex was solvated in a rectangular parallelepiped water box with a 20.0 Å water cap using the TIP3P explicit solvent model. The system was then neutralized by adding Cl^−^ counterions. Prior to MD, a two-step minimization was performed. In the first stage, a 100 kcal/(mol·Å^2^) position restraint was imposed on the whole complex, allowing only water molecules to relax through 5000 steps of steepest descent followed by conjugate gradient minimization. In the second stage, the entire system was minimized applying a harmonic restraint of 10 kcal/(mol·Å^2^) only to the protein Cα atoms. The simulations were conducted using particle mesh Ewald (PME) electrostatics and periodic boundary conditions. Upon minimization, the heating MD step (0.5 ns) was performed using constraint-volume periodic boundary conditions with temperature rising from 0 to 300 K. Subsequently, 3 ns of equilibration were executed through constant pressure periodic boundary MD. The Langevin thermostat was employed in this step to keep the temperature of the system at the constant value of 300 K. Lastly, a production MD run of 196.5 ns was carried out under constant pressure and temperature conditions. In total, 200 ns of MD simulation were therefore performed, and the resulting trajectory was analyzed through the cpptraj program implemented in AMBER24. During the entire MD simulation, a harmonic force constant of 10 kcal/(mol·Å^2^) was applied to restrain all Cα of the protein. This approach preserved the overall experimental conformation of the protein while still allowing for full side-chain flexibility and ligand adaptation within the binding site, thereby enabling an accurate evaluation of the stability of the ligand–protein interactions.

## 3. Results

### 3.1. Machine Learning Model Generation, Optimization, and Assessment

In order to develop ML models for identifying novel ligands potentially active against CDK9, we constructed a curated dataset by retrieving inhibitory activity data from ChEMBL31 [13], considering both CDK9 as an isolated target and as part of the biologically relevant CDK9/cyclin T1 complex. Only activity values reported with exact quantitative measures were retained. As IC_50_ data represented the majority of available measurements (~80%), they were prioritized for training, while the remaining data types were used for model evaluation. After being subjected to the data curation process, compounds were classified as active and inactive according to two different classification settings, i.e., Lead-Oriented (LO) and Potency-Oriented (PO), and for each scheme, compounds with potency values falling within the corresponding classification thresholds were excluded (see Section 2). This decision aimed to avoid ambiguity near the classification boundary, where small fluctuations in measured activity could result in a switched class assignment. The two classification approaches resulted in two parallel workflows, which were carried out simultaneously, enabling a comparative analysis of model performance under different activity definitions. Due to an initial imbalance favoring the active class observed across all datasets, a two-step enrichment strategy was applied to increase the number of inactive compounds. In the first step, additional inactive molecules retrieved from ChEMBL were included, as detailed in the Section 2. Despite this integration, the datasets remained skewed, prompting the inclusion of an additional set of presumed inactive decoy compounds retrieved from the ZINC20 database [16] to further balance the class distribution. These decoys were selected to resemble drug-like molecules while being presumed inactive against CDK9. All added compounds underwent the same preprocessing protocol, and dataset integrity was ensured by eliminating any overlap between training and test sets. The resulting four datasets, two per classification scheme, were fully balanced in terms of classes, as summarized in Table 1.

The generated models were obtained by representing the training set compounds through seven distinct molecular representations, including three types of molecular fingerprints (i.e., RDKit, Morgan, and PubChem fingerprint), and a set of 208 numerical descriptors calculated with RDKit [17]. To leverage complementary information from both structural patterns and physicochemical properties, we also constructed three hybrid representations by concatenating each fingerprint type with RDKit descriptors. These encodings were then used as input for five different ML algorithms: RF, SVM, KNN, GP, and MLP. By systematically combining all representations with all algorithms, a total of 70 models were generated, 35 for each activity threshold scheme. Every model combination was subjected to a dedicated hyperparameter optimization process aimed at maximizing predictive performance. Subsequently, each optimized model underwent a 5-fold CV procedure to assess its robustness across different data partitions. Results of the CV analysis are reported for each model in Tables S1–S5, using MCC as a performance metric. Overall, the CV performance indicates high stability across different data splits, as reflected by the low standard deviation values observed for most models. Interestingly, both the most complex (MLP, Table S5) and the simplest (KNN, Table S4) algorithms showed overall slightly lower performance compared to more traditional ML methods such as RF, SVM, and GP (Tables S1–S3). This trend may be partially explained by the nature of the dataset, which, although of moderate size, may not provide sufficient statistical power for deep learning architectures like MLP to generalize effectively. Deep learning models typically require large amounts of data to achieve optimal performance, while with datasets limited in size, they are prone to overfitting and instability. This is also reflected by the observed standard deviation for MLP-based models (Table S5), which appears to be consistently higher than the one observed for models based on other architectures (Tables S1–S4). On the other hand, KNN may struggle with the complexity and noise of chemical space due to its reliance on local similarity measures, which are more sensitive to class overlap and uneven data distributions. These limitations make intermediate-complexity models, such as RF, SVM, and GPC, better suited to effectively capture informative patterns in the datasets used in this study.

From the molecular representation perspective, hybrid encodings generally performed comparably to standard fingerprints, as particularly evident in the case of KNN models (Table S4). In certain instances, the addition of physicochemical descriptors to fingerprints slightly improved model performance. For example, the SVM model using HybridRR achieved a marginal improvement (ΔMCC = +0.07) over the corresponding model based solely on RDKit fingerprint (Table S2). However, this trend was not consistent across all model families: in some cases, especially when using the Morgan fingerprint, the integration of descriptors led to a reduction in performance, suggesting that the additional information may introduce noise or redundancy rather than a useful signal, depending on the algorithm and representation. As an example, PO models employing the RF and SVM algorithms (Tables S1 and S2) exhibited a decrease in performance, with ΔMCC values of −0.02 and −0.05, respectively. Regarding the data labeling strategy, models developed under the LO approach generally achieved higher MCC values across different algorithms, with the effect being more pronounced for MLP, KNN, and GP models (Tables S3–S5), and less marked for SVM and RF (Tables S1 and S2).

Upon CV analysis, each ML model was derived using the optimal hyperparameter setting previously identified and trained on the corresponding training set. To evaluate the generalization capabilities of each model on unseen data, thereby simulating the real scenario where models are required to predict the activity of unknown compounds, the threshold-specific test sets were used. Models were subsequently ranked based on the MCC values obtained during this step (Tables S6 and S7), and the best-performing one for each classification scheme was identified. For the PO approach, the highest MCC was achieved by the GP-RDKit fingerprint model (MCC = 0.71, Table S6), while for the LO scheme, the RF-Morgan fingerprint model yielded the best performance with an MCC of 0.61 (reported in Table S7). For the LO scheme, the Morgan fingerprint representation proved particularly effective, characterizing most top-ranking models, as reported in Table S7. Conversely, models relying on RDKit descriptors generally showed lower performance. Consistent with CV results, models based on the simplest and the most complex algorithms (KNN and MLP, respectively) performed generally worse than the others. A similar trend was observed for the PO models (Table S6), where most KNN- and MLP-based models were ranked among the lowest-performing ones, often combined with RDKit descriptors. Interestingly, the GP-RDKit fingerprint model of the PO classification scheme achieved the overall highest MCC (Table S6), reflecting the best capability to correctly predict both classes, and was therefore selected to guide the VS campaign.

### 3.2. Virtual Screening

**Figure 1:**
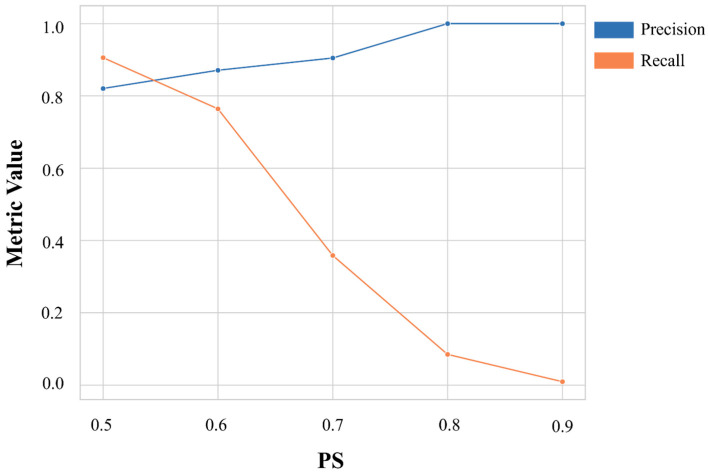
Precision and recall values achieved at different probability scores (PS) thresholds. Precision is reported in blue, while recall is reported in orange.

Building on the results of the previous analysis, a VS study was carried out to identify novel potential inhibitors of the CDK9 protein. For this purpose, the best-performing model (GP-RDKit fingerprint) was employed. The screening was conducted on compounds from the AMS library, which was curated following the same procedure applied to the training and test sets. After curation, the over 7 million compounds obtained were represented as RDKit fingerprints and submitted to the selected model to identify potential hit compounds. To guide the selection of the most promising candidates, all compounds predicted as active with a PS above 0.50 (411,881 molecules) were retrieved and ranked accordingly. To define an appropriate PS threshold for compound selection, the model performance on the test set was examined across values ranging from 0.50 to 0.90, focusing on how precision and recall varied (Figure 1).

At a threshold of 0.8, the model achieved perfect precision, indicating a high confidence in the predicted actives and no false positive predictions. Based on this outcome, the 93 VS hits with PS ≥ 0.8 were prioritized for further filtering. To identify a representative subset of compounds capturing a broad chemical diversity within the 93 predicted hits, hierarchical agglomerative clustering was applied. Compounds were represented using RDKit fingerprint, and pairwise similarity was computed using the Tanimoto coefficient, a standard metric in cheminformatics for comparing molecular fingerprints (see Section 2). As a result, 21 clusters were identified by applying a distance cutoff of 0.30, a threshold informed by a previously reported protocol [10] and verified through visual inspection to be effective in the present scenario. From each cluster, the most promising compound was selected based on the associated PS and its commercial availability at the time of purchase. This filtering process led to the selection of 14 structurally diverse compounds, which were then purchased and submitted to enzymatic assays for experimental validation.

### 3.3. Enzymatic Assays

The 14 compounds selected from the VS campaign were subjected to enzymatic assays to evaluate their inhibitory activity against CDK9. The outcomes of this experimental validation are summarized in Table 2.

Around 30% of the tested compounds (4 out of 14) showed inhibitory activity below 100 µM, with compounds **1**, **2**, and **3** showing IC_50_ values below 50 µM. Most notably, two promising CDK9 inhibitors were identified, namely compounds **1** and **2**, which demonstrated inhibitory activity in the low micromolar range (with IC_50_ values of 3.51 µM and 16.80 µM, respectively). Consequently, these two compounds were prioritized for further investigation. To better assess their inhibitory effects in a more physiologically relevant context, both candidates were advanced to cellular assays.

### 3.4. Antiproliferative Assays

**Figure 2:**
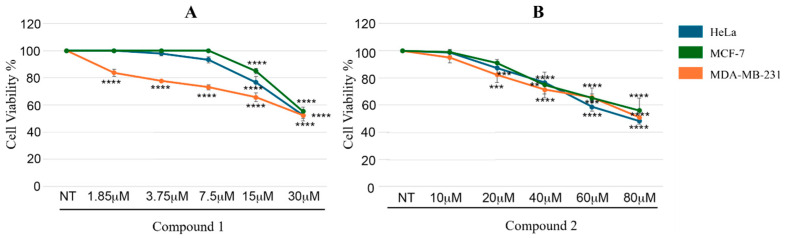
Antiproliferative effect measured as cell viability percentage achieved at different concentrations of compound **1** (**A**) and **2** (**B**) on the three selected cell lines. Results are presented as the mean ± standard deviation (SD) from three independent experiments. Statistically significant differences are indicated as ** *p* < 0.01, *** *p* < 0.001 and **** *p* < 0.0001.

To evaluate the potential anticancer activity of compounds **1** and **2**, we initially performed cell viability assays on a panel of representative tumor cell lines, including HeLa, MCF-7, and MDA-MB-231, which express different molecular characteristics [26]. Treatment with increasing concentrations of the two inhibitors revealed a clear dose-dependent reduction in cell viability. Specifically, compound **1** consistently displayed stronger antiproliferative activity across all tested cell lines (Figure 2A). In contrast, as reported in Figure 2B, compound **2** was less effective at comparable concentrations; however, when administered at higher doses, it demonstrated a significant ability to decrease cell viability, although with lower efficacy than compound **1**.

**Figure 3:**
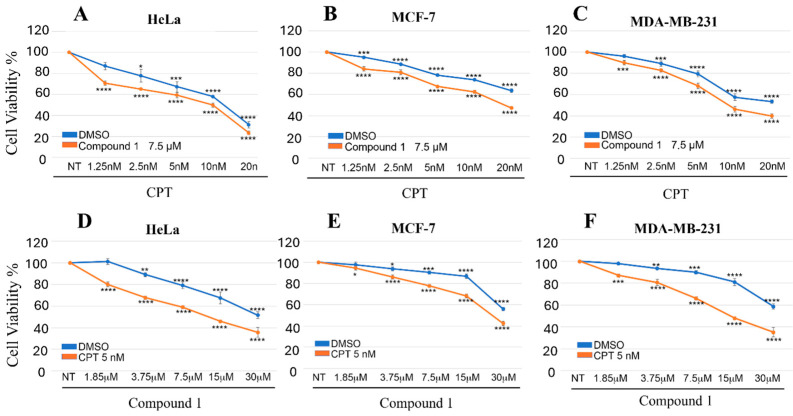
Cell viability assay of HeLa, MCF-7, and MDA-MB-231 cells treated with indicated doses of CPT and compound **1**, alone or in combination, for 72 h. * *p* < 0.5, ** *p* < 0.01, *** *p* < 0.001 and **** *p* < 0.0001. Results are presented as the mean ± SD from three independent experiments: (**A**–**C**) report the results obtained with the first experimental setting, where the concentration of compound **1** was held fixed; (**D**–**F**) refer to the results obtained with the second setting, in which CPT was kept at a fixed concentration.

These findings are consistent with the in vitro enzymatic assays, in which compound **1** displayed approximately a five-fold higher potency than compound **2**. Taken together, the obtained results indicate that compound **1** possesses not only stronger inhibitory activity but also greater therapeutic potential. Therefore, compound **1** was prioritized as the most promising candidate for further studies, with particular attention to its potential use in combination with conventional chemotherapy. Accordingly, we investigated whether CDK9 inhibition by compound **1** could enhance the activity of established anticancer agents. In particular, we focused on Camptothecin (CPT), a topoisomerase I inhibitor widely used in cancer therapy [27]. To explore its potential interaction with compound **1**, we first tested an intermediate concentration of the inhibitor (7.5 µM) in combination with a range of CPT doses (1.25–20 nM). This analysis (Figure 3A–C) provided an overview of the interaction profile and guided the selection of optimal conditions for subsequent experiments. On this basis, a fixed dose of CPT (5 nM) was chosen as a reference, as it produced a detectable cytotoxic effect while still allowing the evaluation of any additive or synergistic contribution from compound **1**. Under these conditions, we next tested an extended range of compound **1** concentrations (1.85–30 µM), which enabled a more detailed characterization of the combined treatment effects (Figure 3D–F).

As shown in Figure 3A–C, compound **1** consistently potentiated the cytotoxic activity of CPT across all tested concentrations relative to the DMSO control. On the other hand, as illustrated in Figure 3D–F, the use of a fixed CPT dose further amplified the cytotoxic effect of compound **1**, demonstrating the presence of an additive interaction between the two agents in all examined cell lines.

To further confirm that the cytotoxic effects of compound **1** are mediated through CDK9 inhibition, we performed cell viability assays in HeLa cells in which CDK9 expression was selectively reduced by siRNA-mediated knockdown, using non-targeting siRNA (siCTR) as a control. Cells with reduced CDK9 expression displayed higher survival upon treatment with compound **1** compared to control cells (results reported in Figure S2). This finding suggests that the presence of CDK9 contributes to the cytotoxic response induced by compound **1**. Consistently, when CDK9 is depleted, the overall impact of compound **1** is reduced. These results support the conclusion that the activity of compound **1** relies on CDK9 inhibition, thereby strengthening the evidence for CDK9 as the primary mediator of the herein identified inhibitory effect.

To gain deeper insight into the mechanism underlying the combined effect of compound **1** and CPT, we examined whether the former could influence the extent of DNA damage induced by CPT. Immunofluorescence analysis of γ-H2AX foci, a well-established marker of double-strand breaks [28], revealed a stronger DNA damage response in cells treated with the drug combination compared to CPT alone, as reported in Figure S3. These findings suggest that compound **1** potentiates the genotoxic effect of CPT. Furthermore, as CPT primarily induces double-strand breaks during the S phase, which are normally repaired through homologous recombination (HR), we next asked whether compound **1** might interfere with this repair pathway. To address this, HeLa cells were pretreated with compound **1** or DMSO for one hour, followed by a two-hour exposure to CPT, and the phosphorylation of RPA32 at S4/8, a marker of HR activity [29], was assessed by immunofluorescence. As shown in Figure S4, no significant changes in pRPA32 signal were observed upon treatment with compound **1**, indicating that HR processivity remained unaltered. Collectively, these results indicate that compound **1** potentiates CPT-induced cytotoxicity by enhancing DNA damage, without impairing HR repair.

### 3.5. Molecular Modeling Studies

**Figure 4:**
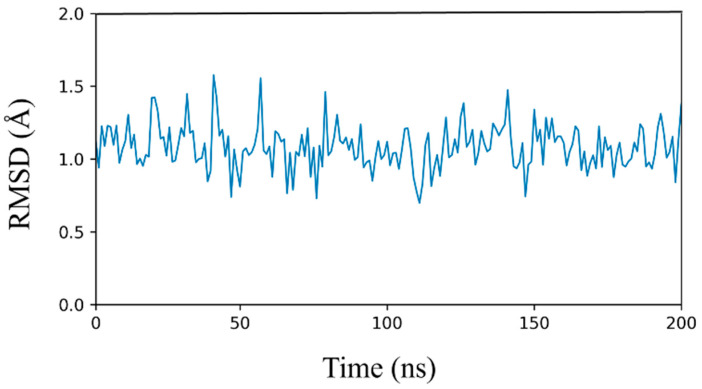
Analysis of MD simulation for compound **1**.

To propose a potential binding mode of compound **1** within the CDK9 active site, we carried out a molecular docking study followed by MD simulation using the X-ray structure of CDK9 (PDB ID: 6GZH [23]). Initially, compound **1** was subjected to docking analysis using GOLD software version 5.1 with the GoldScore fitness function. Clustering of the resulting docking poses with a root mean squared deviation (RMSD) cutoff of 2.0 Å (see Section 2) generated only one reliable binding model for the ligand, characterized by a highly predominant population (97%), and showing at least one hydrogen bond within the hinge region of the protein. This pose was then subjected to 200 ns of MD simulation to assess the stability of the predicted binding conformation and to examine the key interactions with CDK9. Throughout the simulation, the ligand maintained a stable orientation, with an average RMSD relative to the initial docking pose of approximately 1 Å (Figure 4).

**Figure 5:**
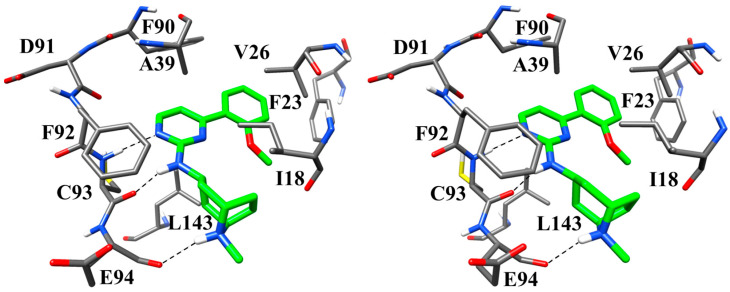
Stereoscopic representation of the minimized average structure of compound **1** in complex with CDK9. Hydrogen bonds are represented as black dashed lines. Protein residues surrounding the ligand, which is represented in green, are shown as grey sticks.

The remarkable stability of the ligand disposition, as well as its interactions with the protein binding site, throughout the whole simulation, confirmed the reliability of the predicted binding mode of compound **1** into CDK9. Figure 5 shows the minimized average structure of CDK9 bound to compound **1** obtained from the last 100 ns of MD simulation, whereas Figure S5 provides a molecular surface representation of the binding site along with compound **1**, highlighting the shape complementarity between the ligand and the surrounding pocket. The predicted binding mode of the ligand is mainly stabilized by three hydrogen bonds involving two residues of the protein hinge region. In particular, residue C93 is involved in two distinct hydrogen bonding interactions. The first occurs between its backbone nitrogen and the pyrimidine nitrogen of the ligand, while the second involves the backbone oxygen of the residue and the NH linker connecting the tropane and pyrimidine moieties of the inhibitor. Additionally, the tropane core further stabilizes the ligand binding through the formation of a third hydrogen bond with the backbone oxygen of residue E94. Beyond these polar interactions, the tropane ring engages in hydrophobic contacts mainly with residue I18, whereas the pyrimidine ring establishes hydrophobic interactions with A39 and L143. Furthermore, the methoxyphenyl ring of the ligand is accommodated within a hydrophobic pocket defined by residues F23, V26, and F90, forming notable hydrophobic interactions with V26 and π–π stacking interactions with F23 and F90, collectively contributing to the stabilization of the ligand–protein complex.

## 4. Discussion

CDK9 plays a central role in transcriptional elongation and DNA repair and exerts its biological activity primarily in complex with cyclin T1, forming the catalytic core of P-TEFb. Beyond its well-established role in cancer, CDK9 has also been implicated in several other pathological conditions characterized by transcriptional dysregulation, including inflammatory, autoimmune, and cardiovascular diseases, underscoring its relevance as a multifaceted therapeutic target across diverse biomedical areas.

Driven by the objective of exploiting the power of ML to identify novel and promising compounds with high therapeutic potential as CDK9 inhibitors, we first collected and curated bioactivity data for both CDK9 alone and the CDK9/cyclin T1 complex. This strategy ensured that the models were both trained on biologically meaningful information and built upon a sufficiently large dataset, two key requirements for obtaining robust and reliable ML models. Upon data standardization, two different classification schemes (LO and PO, as previously detailed) were defined with the aim of exploring model performance under varying levels of pharmacological stringency. The LO scheme was adapted from a previous study conducted on a closely related kinase target [11], where these cutoffs proved effective. Furthermore, in VS contexts, micromolar or sub-micromolar potency is generally associated with compounds of interest for further optimization [30]. However, considering the distribution of the collected bioactivity data (Figure S1), which revealed that most compounds tested against CDK9 exhibited strong potency, a second and more selective classification scheme (PO) was also implemented. The purpose of setting higher potency thresholds with a broader window between them was to train an alternative family of models using highly potent compounds, thereby focusing the learning process on distinguishing molecules with strong inhibitory potential from weaker or inactive ones.

The derived datasets were embedded in seven different molecular representations to evaluate how diverse structural encodings could influence model performance. In addition to standard molecular fingerprints and numerical descriptors, hybrid representations combining them were also generated. This design aimed to explore whether integrating the binary substructure information captured by fingerprints with the continuous physicochemical properties described by molecular descriptors could provide a more informative basis for training predictive ML models. Nevertheless, the results obtained showed that the combination of the two representations led to slight improvements only for certain models, while in others, it likely introduced noise rather than useful information, resulting in a decrease in predictive performance compared with non-hybrid representations. In a broader perspective, this observation underscores the importance of developing multiple models based on different algorithms and molecular encodings, since it is not possible to determine a priori which combination will yield the best performance for a given predictive task. Subsequently, considering the importance of evaluating models in terms of their applicability and generalization to novel compounds, all developed models were further validated using the corresponding test set. The best-performing model was identified as the GP-RDKit fingerprint model developed under the PO approach. In addition to its superior performance, this model was trained using a stricter activity threshold, potentially enhancing its ability to prioritize highly promising candidates. Collectively, these aspects supported its selection as the reference model for the in silico screening campaign. Additionally, the chemical space used to train and validate this model was characterized by projecting RDKit fingerprints for the PO dataset onto the first two principal components obtained from the PCA analysis. This projection (Figure S6) displays a broad dispersion of structures, reflecting substantial structural variability and a high degree of chemical diversity within the dataset.

The 14 most promising candidates resulting from the VS were purchased and subjected to enzymatic assays to experimentally validate the ML-based workflow. The obtained results confirmed the effectiveness of the proposed approach, as two candidates, namely compounds **1** and **2**, displayed low-micromolar inhibitory potency toward CDK9, a level of activity generally regarded as highly promising in early stages of drug discovery. These findings further illustrate the value of a rapid, data-driven, and cost-efficient computational strategy capable of identifying biologically active molecules without the need for extensive experimental screening, in accordance with previous evidence supporting the effectiveness of such approaches.

Considering the promising inhibitory activity of these two compounds, their potential therapeutic relevance was subsequently investigated through biological assays conducted in cellular models. Compound **1**, in particular, emerged as the most promising candidate, showing consistent antiproliferative effects across multiple cancer cell lines. In light of the growing clinical evidence that rational drug combinations can enhance therapeutic efficacy and help overcome resistance mechanisms, we next evaluated its interaction with CPT, a chemotherapeutic agent commonly employed in cancer treatment. The results revealed an additive effect between CDK9 inhibition by compound **1** and CPT treatment, suggesting that the two agents act cooperatively to increase cytotoxicity. Notably, the magnitude of this response varied among the tested cell lines, indicating that the characteristic genetic and molecular backgrounds of each cell type could influence their combined activity. Such variability is particularly relevant in the context of precision oncology and personalized therapy, where the design and selection of cancer treatments increasingly depend on the patient’s specific genetic profile. Finally, additional experimental assays were performed to better characterize the mechanism underlying this combined effect, showing that the cytotoxic activity of compound **1** is dependent on CDK9 inhibition and that its co-administration with CPT amplifies CPT-induced DNA damage without compromising the efficiency of HR repair.

As a final step, a computational investigation combining molecular docking and MD simulation was carried out to provide a structural rationale for the observed biological activity. The proposed binding mode, deemed reliable in light of its remarkable stability, revealed that compound **1** forms a consistent network of hydrogen bonds and hydrophobic interactions with key residues within the CDK9 binding pocket.

Within this framework, previously reported computational efforts offer useful points of comparison. Lately, several computational strategies have been successfully applied to identify novel CDK9 inhibitors [31,32,33,34,35] and to characterize the activity profiles of small molecules against diverse kinase targets [12,36]. Moreover, ML-based VS has repeatedly demonstrated its ability to efficiently prioritize promising compounds with reduced experimental cost and time across multiple target classes, including kinases [10,11,37]. However, despite these advances, the specific case of CDK9 still presents unmet needs. Existing studies contribute valuable data and identify new molecules without, however, providing accessible predictive tools, ultimately limiting the practical adoption of ML-based methods by the wide scientific community for new discovery efforts. To the best of our knowledge, only one study provides a directly comparable baseline, as it publicly releases an ML classifier model for predicting CDK9 activity [38]. When evaluated on our test set, this model reached an MCC of 0.47, whereas the classifier developed under the PO strategy (GP–RDKit fingerprints), which we used to guide the VS campaign and was made publicly available, achieved an MCC of 0.71. Viewed within the broader scientific landscape, the relevance of the herein presented work becomes evident, as it addresses a persistent gap in a field where accelerating the discovery of new CDK9 inhibitors remains a critical priority. Furthermore, when considered together with the structural insight provided by our computational analysis, which may support future optimization efforts, the overall contribution of this study is further enhanced, underscoring its value in advancing computationally guided strategies for the identification and refinement of novel CDK9 inhibitors.

## 5. Conclusions

CDK9 is a multifunctional kinase regulating key processes from DNA repair to transcriptional elongation. By sustaining the expression of oncogenes and short-lived anti-apoptotic proteins, CDK9 supports cancer cell survival, proliferation, and resistance to conventional therapies. Its overexpression in multiple solid and hematological malignancies is indeed associated with poor prognosis and increased metastatic potential. Moreover, CDK9 plays a role beyond oncology, being implicated in other pathological conditions characterized by transcriptional dysregulation. Therefore, CDK9 represents a highly relevant therapeutic target, as inhibiting its activity can disrupt critical survival pathways and holds promise for the development of novel therapeutic approaches in diseases where transcriptional control is altered.

In this study, a systematic process of data collection, preprocessing, and model optimization was applied to generate a total of seventy ML models by combining different algorithms, molecular representations, and classification thresholds. The performance of all models was evaluated to identify the best predictor, which was then used to conduct a VS campaign aimed at discovering novel CDK9 inhibitors. Enzymatic assays performed on the most promising candidates resulted from the VS revealed that two compounds, namely compounds **1** and **2**, exhibited inhibitory activity in the low micromolar range, with IC_50_ values of 3.51 µM and 16.80 µM, respectively. Considering that inhibitory activity in the low micromolar range is regarded as both encouraging and highly promising at this stage of drug discovery, these two compounds were advanced to cell-based assays on three cancer cell lines, HeLa, MCF-7, and MDA-MB-231, selected to represent different tumor types and genetic contexts. Among them, compound **1** displayed significant cytotoxic activity. Further experiments investigated the effect of compound **1** in combination with CPT. The combined treatment enhanced cytotoxicity compared to single-agent treatments, suggesting a cooperative mechanism that also amplifies DNA damage. Notably, the variable response observed among the tested cell lines indicates that the efficacy of the combination may depend on genetic factors, a concept aligned with the principles of precision oncology and personalized therapy. Finally, structure-based analyses were carried out to predict a plausible binding mode for compound **1**, providing valuable insights that may serve as a foundation for future optimization efforts aimed at developing novel, highly potent CDK9 inhibitors. To encourage transparency and support future studies, the final predictive model was made openly available on GitHub (https://github.com/MMVSL/CDK9Screen, accessed on 25 October 2025), providing researchers with an accessible tool to evaluate the potential inhibitory activity of novel CDK9 candidates.

## Figures and Tables

**Table 1:** Composition of training and test sets for each classification scheme: Lead-Oriented (LO) and Potency-Oriented (PO).

**Lead-Oriented (LO)**			
	**Active**	**Inactive**	**Total**
**Training**	1375	1375	2750
**Test**	279	279	558
**Potency-Oriented (PO)**			
	**Active**	**Inactive**	**Total**
**Training**	447	447	894
**Test**	106	106	212

**Table 2:** Structure and experimental CDK9 inhibitory activity of the tested compounds. Roscovitine was used as a reference inhibitor.

Cpds.	Structure	Experimental IC_50_ (nM)
**1**	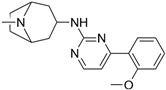	3510
**2**	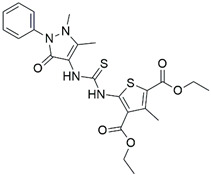	16,800
**3**	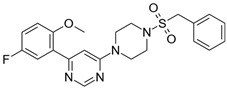	28,600
**4**	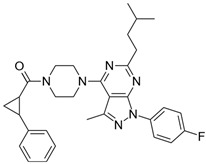	67,200
**5**	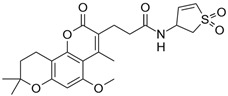	107,000
**6**	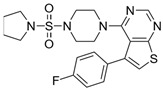	133,000
**7**	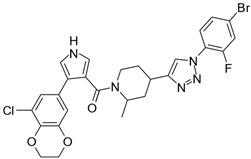	250,000
**8**	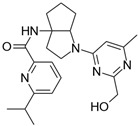	250,000
**9**	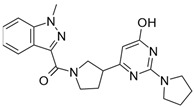	250,000
**10**	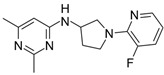	250,000
**11**	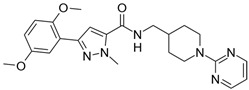	250,000
**12**	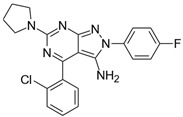	250,000
**13**	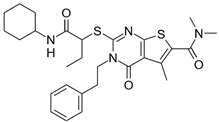	250,000
**14**	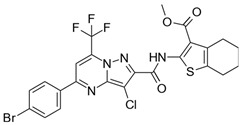	250,000
**Roscovitine**	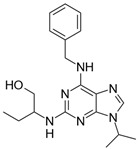	476

## Data Availability

Original microscopy images have been deposited at Zenodo at https://doi.org/10.5281/zenodo.17472637. The ML model developed and used for the identification of novel CDK9 inhibitors was made publicly available as a predictive tool on GitHub (https://github.com/MMVSL/CDK9Screen, accessed on 25 October 2025).

## Supplementary Materials

The following supporting information can be downloaded at: https://www.mdpi.com/article/10.3390/biom16010012/s1, Figure S1: Distribution of activity values for training set compounds; Table S1: Cross validation results for RF-based models; Table S2: Cross validation results for SVM-based models; Table S3: Cross validation results for GP-based models; Table S4: Cross validation results for KNN-based models; Table S5: Cross validation results for MLP-based models; Table S6: Test set performance of PO models; Table S7: Test set performance of LO models; Figure S2: Cell viability assays in HeLa cells transfected with siCTR or siCDK9; Figure S3: Immunofluorescence analysis of γ-H2AX foci; Figure S4: Analysis of HR activity; Figure S5: Molecular surface representation of the CDK9 binding site in complex with compound 1; Figure S6: PCA projection of PO dataset.
