# Supplementary material for: Machine Learning-Based Virtual Screening for the Identification of Novel CDK-9 Inhibitors
Source: Biomolecules. 2025 Dec 20;16(1):12. doi: 10.3390/biom16010012 (PMC12839014; doi:10.3390/biom16010012)
Supplement: Supplementary file 1 [file biomolecules-16-00012-s001.zip › biomolecules-4021449-supplementary.pdf]

# Supporting Information

# Machine Learning-Based Virtual Screening for the Identification of Novel CDK-9 Inhibitors

**Lisa Piazza [1], Clarissa Poles [2,3], Giulia Bononi [1], Carlotta Granchi [1], Miriana Di Stefano [1], Giulio Poli [1], Antonio Giordano [4], Annamaria Medugno [5], Giuseppe Maria Napolitano [6,*], Tiziano Tuccinardi [1,7,*] and Luigi Alfano [8]**

[1] Department of Pharmacy, University of Pisa, 56126 Pisa, Italy; lisa.piazza@phd.unipi.it (L.P.); giulia.bononi@unipi.it (G.B.); carlotta.granchi@unipi.it (C.G.); miriana.distefano@farm.unipi.it (M.D.S.); giulio.poli@unipi.it (G.P.)
[2] Genomics and Experimental Medicine Program, Scuola Superiore Meridionale (SSM, School of Advanced Studies), Via Mezzocannone 4, 80078 Napoli, Italy; c.poles@tigem.it
[3] Telethon Institute of Genetics and Medicine, Via Campi Flegrei 34, 80078 Napoli, Italy
[4] Sbarro Institute for Cancer Research and Molecular Medicine, Center for Biotechnology, College of Science and Technology, Temple University, Philadelphia, PA 19122, USA; antonio.giordano@temple.edu
[5] Department of Medical Biotechnologies, University of Siena, 53100 Siena, Italy; a.medugno1@studenti.unisi.it
[6] Clinical and Translational Oncology Program, Scuola Superiore Meridionale (SSM, School of Advanced Studies), University of Naples Federico II, 80131 Napoli, Italy
[7] Consorzio Interuniversitario Nazionale per la Scienza e Tecnologia dei Materiali (INSTM), 50121 Firenze, Italy
[8] Department of Breast and Thoracic Oncology, Istituto Nazionale Tumori-IRCCS-Fondazione G. Pascale, 80131 Napoli, Italy; l.alfano@istitutotumori.na.it
* Correspondence: g.napolitano@ssmeridionale.it (G.M.N.); tiziano.tuccinardi@unipi.it (T.T.)

## Table of Contents

**Figure S1** Distribution of activity values for training set compounds S2

**Table S1** Cross-validation results for RF-based models S2

**Table S2** Cross-validation results for SVM-based models S3

**Table S3** Cross-validation results for GP-based models S3

**Table S4** Cross-validation results for KNN-based models S4

**Table S5** Cross-validation results for MLP-based models S4

**Table S6** Test set performance of PO models S5

**Table S7** Test set performance of LO models S5

**Figure S2** Cell viability assays in HeLa cells transfected with siCTR or siCDK9 S6

**Figure S3** Immunofluorescence analysis of $\gamma$-H2AX foci S7

**Figure S4** Analysis of HR activity S8

**Figure S5** Molecular surface representation of the CDK9 binding site in complex with compound 1 S9

**Figure S6** PCA projection of PO dataset S9

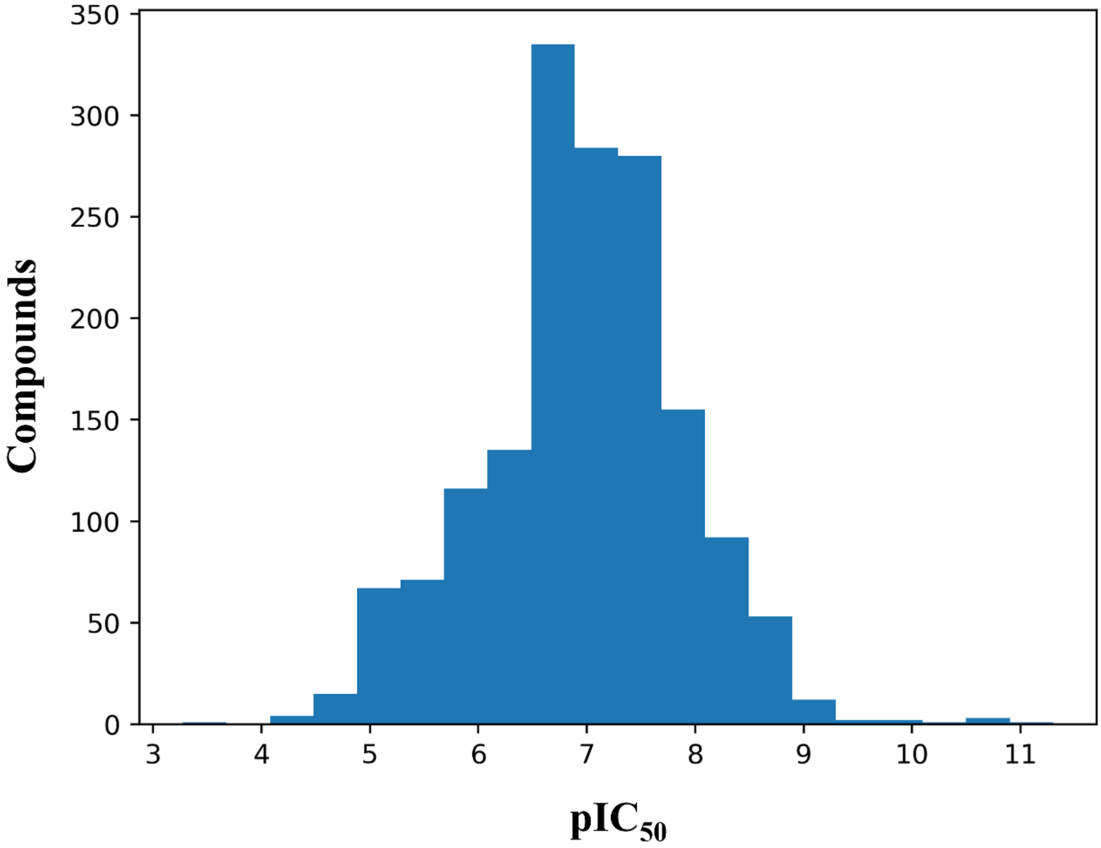


**Figure S1.** Distribution of activity values, expressed as $pIC_{50}$, of training compounds prior to classification and further integration with inactive decoys.

**Table S1.** Cross validation performance, evaluated through MCC, for RF-based models. Models derived according to the Potency-Oriented (PO) and Lead-Oriented (LO) approaches are reported.

| Approach | Molecular Representation | MCC |
|---|---|---|
| **PO** | Morgan fingerprint | 0.87 +/- 0 |
| **PO** | RDKit fingerprint | 0.87 +/- 0 |
| **PO** | PubChem fingerprint | 0.88 +/- 0 |
| **PO** | RDKit Descriptors | 0.88 +/- 0 |
| **PO** | HybridMR | 0.85 +/- 0.01 |
| **PO** | HybridRR | 0.83 +/- 0.05 |
| **PO** | HybridPR | 0.87 +/- 0 |
| **LO** | Morgan fingerprint | 0.9 +/- 0.01 |
| **LO** | RDKit fingerprint | 0.89 +/- 0.01 |
| **LO** | PubChem fingerprint | 0.89 +/- 0 |
| **LO** | RDKit Descriptors | 0.88 +/- 0.02 |
| **LO** | HybridMR | 0.91 +/- 0 |
| **LO** | HybridRR | 0.92 +/- 0.01 |
| **LO** | HybridPR | 0.92 +/- 0 |

**Table S2.** Cross validation performance, evaluated through MCC, for SVM-based models. Models derived according to the Potency-Oriented (PO) and Lead-Oriented (LO) approaches are reported.

| Approach | Molecular Representation | MCC |
|---|---|---|
| **PO** | Morgan fingerprint | 0.88 +/- 0 |
| **PO** | RDKit fingerprint | 0.8 +/- 0.02 |
| **PO** | PubChem fingerprint | 0.8 +/- 0.02 |
| **PO** | RDKit Descriptors | 0.87 +/- 0 |
| **PO** | HybridMR | 0.83 +/- 0.03 |
| **PO** | HybridRR | 0.87 +/- 0 |
| **PO** | HybridPR | 0.82 +/- 0.05 |
| **LO** | Morgan fingerprint | 0.92 +/- 0 |
| **LO** | RDKit fingerprint | 0.88 +/- 0.02 |
| **LO** | PubChem fingerprint | 0.89 +/- 0.01 |
| **LO** | RDKit Descriptors | 0.89 +/- 0.0 |
| **LO** | HybridMR | 0.92 +/- 0.02 |
| **LO** | HybridRR | 0.89 +/- 0.02 |
| **LO** | HybridPR | 0.88 +/- 0.0 |

**Table S3.** Cross validation performance, evaluated through MCC, for GP-based models. Models derived according to the Potency-Oriented (PO) and Lead-Oriented (LO) approaches are reported.

| Approach | Molecular Representation | MCC |
|---|---|---|
| **PO** | Morgan fingerprint | 0.76 +/- 0 |
| **PO** | RDKit fingerprint | 0.88 +/- 0 |
| **PO** | PubChem fingerprint | 0.81 +/- 0.06 |
| **PO** | RDKit Descriptors | 0.86 +/- 0.03 |
| **PO** | HybridMR | 0.84 +/- 0.02 |
| **PO** | HybridRR | 0.81 +/- 0.05 |
| **PO** | HybridPR | 0.8 +/- 0.06 |
| **LO** | Morgan fingerprint | 0.89 +/- 0.02 |
| **LO** | RDKit fingerprint | 0.89 +/- 0.02 |
| **LO** | PubChem fingerprint | 0.89 +/- 0.0 |
| **LO** | RDKit Descriptors | 0.9 +/- 0.02 |
| **LO** | HybridMR | 0.9 +/- 0.02 |
| **LO** | HybridRR | 0.89 +/- 0.02 |
| **LO** | HybridPR | 0.89 +/- 0.01 |

**Table S4.** Cross validation performance, evaluated through MCC, for KNN-based models. Models derived according to the Potency-Oriented (PO) and Lead-Oriented (LO) approaches are reported.

| Threshold Approach | Molecular Representation | MCC |
|---|---|---|
| **PO** | Morgan fingerprint | 0.84 +/- 0.06 |
| **PO** | RDKit fingerprint | 0.73 +/- 0 |
| **PO** | PubChem fingerprint | 0.74 +/- 0.01 |
| **PO** | RDKit Descriptors | 0.82 +/- 0 |
| **PO** | HybridMR | 0.85 +/- 0.01 |
| **PO** | HybridRR | 0.75 +/- 0.04 |
| **PO** | HybridPR | 0.78 +/- 0.02 |
| **LO** | Morgan fingerprint | 0.91 +/- 0.01 |
| **LO** | RDKit fingerprint | 0.76 +/- 0.07 |
| **LO** | PubChem fingerprint | 0.83 +/- 0.02 |
| **LO** | RDKit Descriptors | 0.85 +/- 0.01 |
| **LO** | HybridMR | 0.91 +/- 0 |
| **LO** | HybridRR | 0.77 +/- 0.05 |
| **LO** | HybridPR | 0.85 +/- 0.0 |

**Table S5.** Cross validation performance, evaluated through MCC, for MLP-based models. Models derived according to the Potency-Oriented (PO) and Lead-Oriented (LO) approaches are reported.

| Approach | Molecular Representation | MCC |
|---|---|---|
| **PO** | Morgan fingerprint | 0.76 +/- 0.06 |
| **PO** | RDKit fingerprint | 0.58 +/- 0.29 |
| **PO** | PubChem fingerprint | 0.67 +/- 0.15 |
| **PO** | RDKit Descriptors | 0.70 +/- 0.07 |
| **PO** | HybridMR | 0.78 +/- 0.07 |
| **PO** | HybridRR | 0.77 +/- 0.06 |
| **PO** | HybridPR | 0.73 +/- 0.08 |
| **LO** | Morgan fingerprint | 0.89 +/- 0.01 |
| **LO** | RDKit fingerprint | 0.64 +/- 0.33 |
| **LO** | PubChem fingerprint | 0.82 +/- 0.05 |
| **LO** | RDKit Descriptors | 0.86 +/- 0.02 |
| **LO** | HybridMR | 0.89 +/- 0.01 |
| **LO** | HybridRR | 0.83 +/- 0.05 |
| **LO** | HybridPR | 0.84 +/- 0.02 |

**Table S6.** Performance, expressed as MCC, achieved by each PO model on the corresponding test set. Rows correspond to molecular representations and columns to ML algorithms; each cell reports the MCC obtained from the combination of a given representation and algorithm. The molecular representation, algorithm, and MCC value of the best-performing model are shown in bold.

| | RF | SVM | KNN | **GP** | MLP |
|---|---|---|---|---|---|
| Morgan fingerprint | 0.67 | 0.64 | 0.51 | 0.61 | 0.63 |
| PubChem fingerprint | 0.51 | 0.60 | 0.44 | 0.64 | 0.56 |
| **RDKit fingerprint** | 0.63 | 0.68 | 0.46 | **0.71** | 0.64 |
| RDKit Descriptor | 0.05 | 0.49 | 0.28 | 0.49 | 0.49 |
| HybridMR | 0.64 | 0.60 | 0.42 | 0.59 | 0.48 |
| HybridPR | 0.56 | 0.62 | 0.43 | 0.60 | 0.47 |
| HybridRR | 0.53 | 0.66 | 0.48 | 0.67 | 0.68 |

**Table S7.** Performance, expressed as MCC, achieved by each LO model on the corresponding test set. Rows correspond to molecular representations and columns to ML algorithms; each cell reports the MCC obtained from the combination of a given representation and algorithm. The molecular representation, algorithm, and MCC value of the best-performing model are shown in bold.

| | **RF** | SVM | KNN | GP | MLP |
|---|---|---|---|---|---|
| **Morgan fingerprint** | **0.61** | 0.50 | 0.42 | 0.48 | 0.50 |
| PubChem fingerprint | 0.44 | 0.37 | 0.27 | 0.40 | 0.23 |
| RDKit fingerprint | 0.46 | 0.44 | 0.42 | 0.41 | 0.41 |
| RDKit Descriptor | 0.08 | 0.35 | 0.12 | 0.27 | 0.20 |
| HybridMR | 0.32 | 0.52 | 0.37 | 0.50 | 0.42 |
| HybridPR | 0.12 | 0.43 | 0.24 | 0.37 | 0.36 |
| HybridRR | 0.33 | 0.45 | 0.37 | 0.43 | 0.29 |

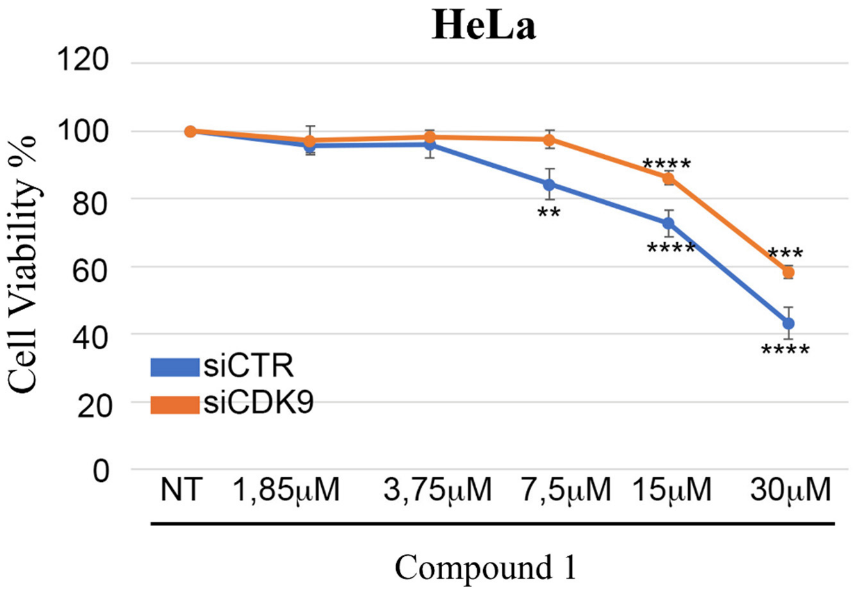


**Figure S2**. HeLa cells were transfected with 100 nM of siCTR or siCDK9. After 24 hours of incubation, the cells were plated for the indicated treatment time points and incubated for an additional 24 hours, followed by treatment with the specified drug concentrations for another 72 hours. Results are presented as the mean ± SD from three independent experiments. *p< 0.5, **p< 0.01, *** p < 0.001 and **** p < 0.0001.

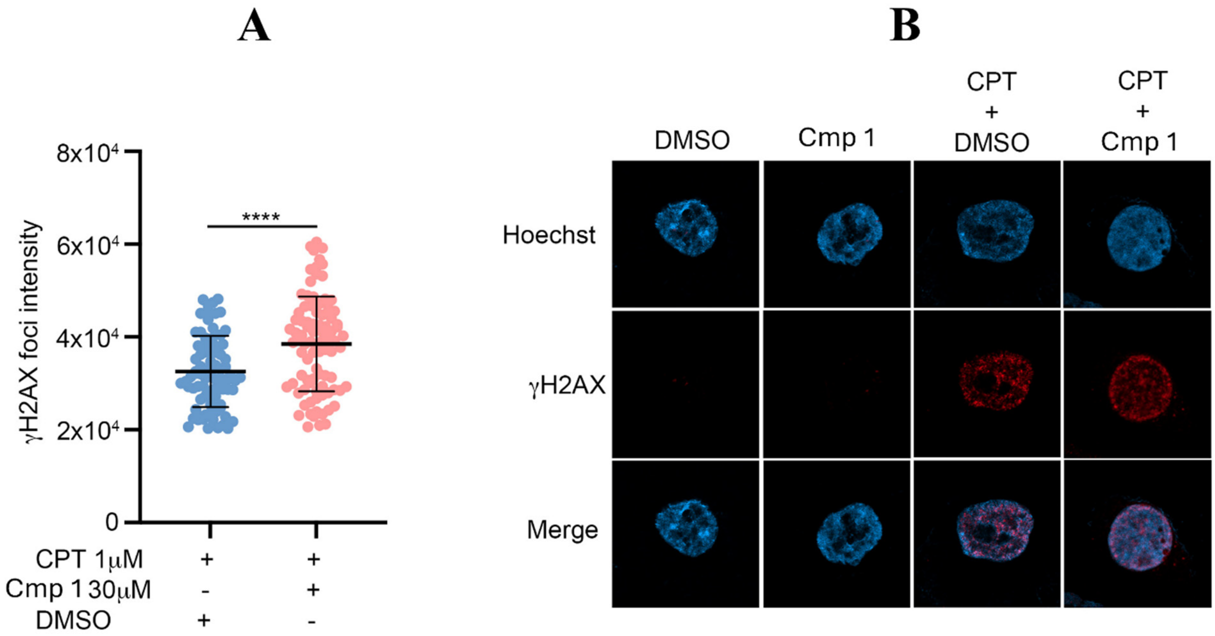


**Figure S3**. A) Immunofluorescence analysis of HeLa cells pretreated with compound **1** (reported as Cmp 1) or DMSO, for one hour followed by incubation with CPT, for additional two hours. Analysis of γ-H2AX foci was performed by using Fiji software. Thirty cells were evaluated per condition. Data are expressed as the mean ± standard deviation (SD) from three separate experiments. Statistical significance was determined using Welch's corrected Student's t-test to account for potential variance differences between samples. **** $p < 0.0001$. B) Illustration of γ-H2AX immunofluorescence of cells treated as in A.

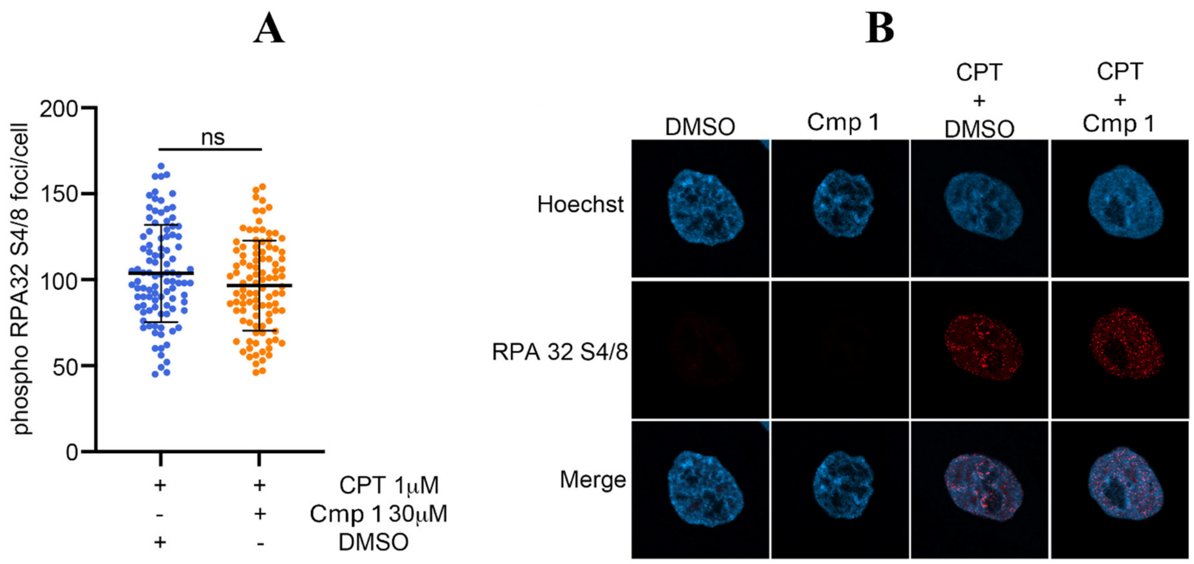


**Figure S4**. A) HeLa cells were pre-treated with compound 1 (reported as Cmp 1) or DMSO for one hour, followed by incubation with CPT. Thirty cells per condition were analyzed from three independent experiments, with results reported as the standard deviation (SD). Statistical analysis was performed using Welch's corrected Student's t-test to account for heteroscedasticity in the data. B) Representative images of cells treated as described in panel A.

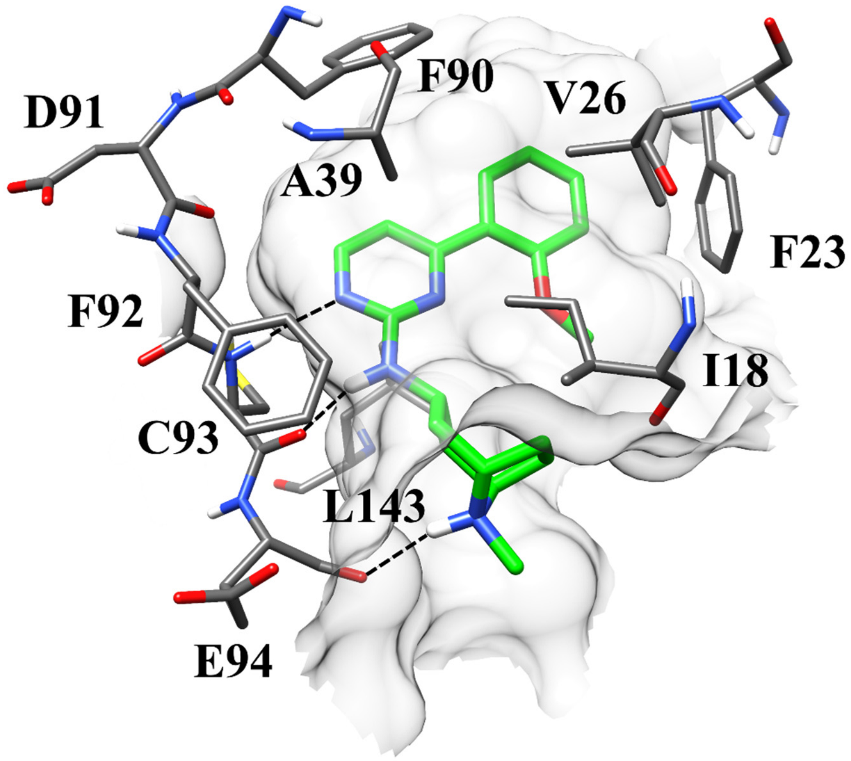


**Figure S5.** Molecular surface representation of the CDK9 binding site in complex with compound **1**. The protein surface is shown in grey and compound **1** is depicted in green. Surrounding residues are represented as grey sticks, and hydrogen bonds are indicated as black dashed lines.

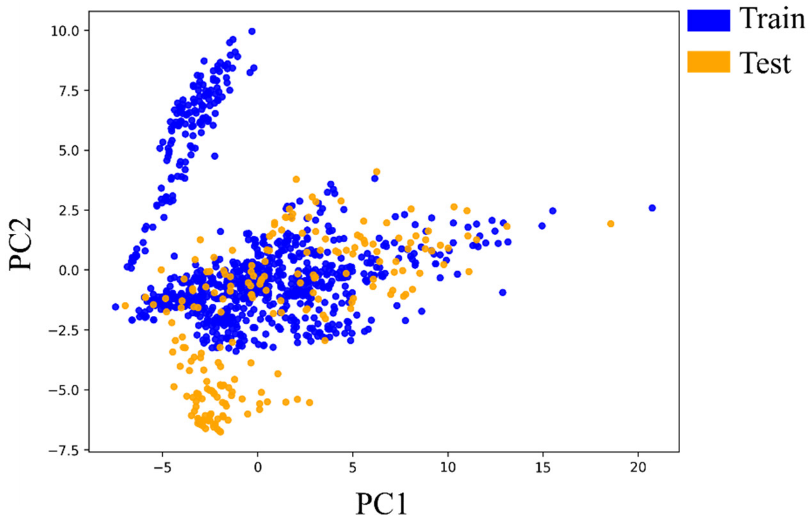


**Figure S6**. Overlaid PCA projection of PO dataset, with training set instances represented by blue dots and test set instances represented by orange dots.
